# Editorial: Alzheimer’s Disease: Original Mechanisms and Translational Impact

**DOI:** 10.3389/fphar.2020.00157

**Published:** 2020-02-26

**Authors:** Cesare Mancuso, Silvana Gaetani

**Affiliations:** ^1^ Fondazione Policlinico Universitario A Gemelli IRCCS, Rome, Italy; ^2^ Institute of Pharmacology, Università Cattolica del Sacro Cuore, Rome, Italy; ^3^ Department of Physiology and Pharmacology “V. Erspamer,” Sapienza University of Rome, Rome, Italy

**Keywords:** Alzheimer’s disease, preclinical studies, drug research and development, neurodegeneration, synaptic plasticity

Alzheimer’s disease (AD) is a neurodegenerative disorder characterized by progressive and irreversible worsening of cognitive functions, inability to perform everyday activities, and mood disorders. Currently, AD is considered the leading cause of dementia and hospitalization of older adults in nursing homes. In the United States, 5.8 million people has been calculated to suffer from AD in 2019, 81% being 75 years or older; the percentage of individuals with AD increases with age, from 3% of people aged 65–74 to 32% of people aged 85 and older. Women are more affected by AD than men (M/F 2/1) probably because of their longer lifespan. Finally, African Americans and Hispanics are about twice likely to develop AD as older Whites (Alzheimer’s Association, 2019). The lack of any updated epidemiologic survey about AD in Europe is quite disappointing; the most accurate analysis dates back 2017 and reveals an estimated prevalence at 5.05% (men 3.31% and women 7.13%) increasing with age (Niu et al., 2017). In Europe, about 3 million people was estimated to suffer from AD (Mayer et al., 2018).

From a pathogenetic viewpoint, the early “amyloid cascade hypothesis”, which considered fibrillar β-amyloid (Aβ) and hyperphosphorylated tau protein (pTau) as the main inducers of the pro-oxidant status and neuroinflammation leading to neuronal death, was definitely challenged (Selkoe and Hardy, 2016). Clinical evidence has clearly demonstrated both the evidence that the amount of senile plaques, containing fibrillar Aβ, does not correlate with the severity of AD and the lack of efficacy of therapies targeting fibrillar Aβ in terms of improvement of cognitive function (Nelson et al., 2012; Penninkilampi et al., 2016; Wang et al., 2017). Over the last few years, soluble Aβ, mainly in the oligomeric form, has been proposed as the toxic species being responsible for the early impairment of synaptic plasticity and neurotransmission occurring in AD (Abdel-Hafiz et al., 2018; Li et al., 2018). Unfortunately, AD begins several years before the onset of symptoms, which become evident when neurodegeneration reaches the point of no return. This is the reason why drugs currently available, such as acetylcholinesterase inhibitors and the N-methyl-D-aspartate (NMDA) receptor antagonist memantine, have only limited symptomatic effects; regrettably, there is not any class of drugs capable of preventing or contrasting the evolution of the disease (Mancuso et al., 2011; Mhillaj et al., 2017). As recently reported by Cummings et al. (2019), 132 drugs are under clinical development for AD and only 28 of them are in phase III; among these latter, nine are anti-amyloid agents, eight are compounds targeting neuropsychiatric symptoms, and only three are antioxidant/neurotransmitter-based therapies.

The aim of this Research Topic is to outline the multifactorial etiology of AD and promising key factors for the development of new and successful therapeutic strategies. The issues addressed in this Research Topic include, among others, the interplay between well-known and novel molecular mechanisms, such as oxidative and neuroinflammatory events leading to synaptic failure, some comorbidities secondary to exaggerated Aβ deposition and the potential therapeutic role for medicinal herbs or drugs to slow-down the progression of AD. This Research Topic, in which several leading experts have provided important contributions, is organized in eight original research articles (including a brief research report), four reviews, and six mini-reviews.

Caruso et al. examined the role of stress and the effects due to the hyperactivation of the hypothalamic-pituitary-adrenal axis as determinants of AD. This review is quite interesting because it focuses the attention on potential lifestyle risk-factors whose elimination could drastically reduce the onset of AD. That said, everybody knows that a stress-free life is an unattainable dream, and the possibility to prevent or contrast AD based on an impossible lifestyle is a vain hope. However, the evidence that specific genetic variants, by reducing either the activity of specific enzymes involved in cortisol degradation (e.g., the 11ß-hydroxysteroid dehydrogenase) or the sensitivity of glucocorticoid receptor to cortisol, might increase or decrease the risk to develop AD, respectively, opens new avenues about the role of tailored medicine for an early diagnosis of dementia. Chemokines and their receptors are widely distributed in both neurons and glial cells and play a pivotal role in neuroinflammation. Zuena et al. highlighted the contribution of prokineticin 2 and its receptors in the pathogenesis of AD; the authors, after a careful analysis of available preclinical data, strong support the hypothesis that the pharmacological antagonism of prokineticin receptors could reduce neurodegeneration, thus including these chemokines in the arena of novel and promising drug targets in AD. The contribution of glial cells, in particular astrocytes, to neuroinflammation is a blooming field of research. In an interesting original research, Grimaldi et al. described the detection of both Aβ and pTau aggregates in the retina of AD patients *vis-à-vis* with neuronal death and detrimental astrocytes and microglial activation. These data, confirm the role of aberrant glial cell activation as a milestone in the pathogenesis of AD, but also suggest the hypothesis to consider retina as an easily accessible window for an early detection of pathological AD hallmarks. Dal Pra et al. described the role of family C G-protein-coupled receptors (GPCR), in particular those expressed by astrocytes, in the onset and progression of AD; furthermore, these authors highlight the role of GPCR as possible drug-targets to challenge neurodegeneration. The effect of aging on astrocyte function was explored by Bronzuoli et al. in a transgenic mouse model of AD (3xTg-AD): the authors demonstrated how aging, rather than AD progression, importantly affects morphology and functions of hippocampal glial cells. These results, novel and provocative, should prompt researchers to further study the role of astrocytes and microglia in both physiological and pathological aging. Recent studies have demonstrated how brain microRNAs participate in multiple aspects of AD pathology: in this regard, Wang, Liu et al. studied microRNA-200a-3p (miR-200a-3p) in transgenic preclinical model of AD (APP/PS1 and SAMP8 mice) and in the blood of AD patients. The authors concluded that this microRNA is neuroprotective through the inhibition of Aβ overproduction *via* suppression of the expression of BACE1 and the synergistic decrease of pTau hyperphosphorylation. The contribution of mitochondria-derived reactive oxygen species in neuronal death is another quite exploited line of research in neurodegenerative diseases. Cenini and Voos provided an updated and exhaustive review about the potential therapeutic efficacy of several agents, including some nutritional antioxidants, to challenge AD by acting at the mitochondrial level. However, the authors concluded that, despite the huge lines of preclinical evince supporting this idea, there is no clinical evidence strong enough to support the hypothesis that mitochondria pharmacological manipulation is currently an option for AD therapy.

The progressive loss of cognitive function in AD subjects was associated to the early impairment in synaptic transmission due to Aβ deposition. Long-term potentiation (LTP) and long-term depression (LTD) are the two most characterized forms of durable synaptic strength, particularly in the hippocampal region, and the magnitude of LTP and LTD is considered as an index of cognitive function in many different experimental conditions. In an interesting mini review, Mango et al. described how LTP and LTD are dysfunctional in several preclinical models of AD. Furthermore, these authors discussed the possible beneficial effects of either investigational agents or non-invasive treatments, such as repetitive transcranial magnetic stimulation and transcranial direct current stimulation, to contrast or slow-down dementia by modulating synaptic plasticity. In a preclinical model of early AD amyloidosis, the McGill-R-Thy1-APP transgenic rat, Qi et al. described the effects of soluble Aβ on synaptic plasticity. According to this study, pre-plaque Aβ mediated an age-dependent inhibition of both LTP and novelty exploration-induced depotentiation in these animals, but only at apical synapses in the CA1 area of hippocampus. The differential susceptibility of plasticity at apical and basal synapses suggests a circuit-selective reduction in the dynamic range of synaptic gain and weakening.

An important aspect that healthcare practitioners must deal with, is the onset of comorbidities in AD patients due to the abnormal Aβ deposition in brain. Cordone et al. provided a detailed review about the occurrence of sleep disturbances in AD subjects as early as Aβ accumulates in the brain. The authors raised the alarm, based on a restricted number of clinical trials, that sleep disruption could lead to deleterious effects on Aβ accumulation in healthy populations. The most useful approach to reduce this risk is to encourage virtuous behavior, such as reducing both the use of psychoactive substances and the time of exposure to light in the evening, practicing physical and social activities, and keeping constant bed and wake times. With regard to pharmacological treatments, melatonin was extensively studied for this purpose, but the final evidence supporting its beneficial role to improve cognitive skills by restoring sleep efficiency is still lacking. Depression is another comorbidity frequently occurring in AD patients, in particular during the preclinical stage, and several lines of evidence have linked soluble Aβ formation with depressive state (Colaianna et al., 2010; Chi et al., 2014). On this regard, Morgese and Trabace summarized the preclinical and epidemiological studies about the role of monoaminergic system impairment as a cause of depression in AD and proposed novel therapeutic approaches based on the modulation of such a neurotransmitter system.

The use of medicinal plants, endowed with antioxidant and neuroprotective features, to contrast neurodegeneration is currently a hot field of research. Angeloni et al. provided a complete mini review on the neuroprotective effects of icariin, a prenylated flavonoid considered as the main bioactive of *Herba epimedii* (a Chinese herbal medicine), in AD. The authors described the pharmacokinetics of icariin as well as the antinflammatory and antioxidant effects in AD. Similarly, Retinasamy et al. described the neuroprotective and nootropic outcomes of *Orthosiphon stamineus*, a medicinal plant abundant in Southeast Asia, in scopolamine-treated rats. Beggiato et al. overviewed the neuropharmacology of *N*-palmitoylethanolamide (PEA), a lipid mediator belonging to the class of the *N*-acylethanolamides and firstly isolated from soy lecithin, egg yolk, and peanut meal. On these bases, both icariin and *Orthosiphon stamineus*, as well as PEA, have been proposed as potential adjuvant therapies in AD subjects.

Over the last few years, many drugs, initially authorized and marketed for the treatment of other diseases, have proven to be potentially effective for the treatment of AD. Ono and Tsuji and Balducci and Forloni put under the spotlight cilostazol and doxycycline, respectively. The first is an antiplatelet drug used for the treatment of intermittent claudication and the second is a wide-spectrum antibacterial drug belonging to the tetracycline family. Both cilostazol and doxycycline were mainly tested in preclinical models of AD and they showed neuroprotective properties in terms of inhibition of soluble Aβ oligomerization and aggregation as well as improvement of antioxidant defense in the brain. Although the efficacy of these two drugs in AD subjects has not been definitively proven (some clinical trials are still ongoing), a possible reposition strategy should be considered for these two agents. LC1405 (7-pyrrolidinethoxy-40-methoxyisoflavone) is a novel potential H_3_ receptor antagonist which has been shown to reduce neurodegenerative damage, ameliorate cholinergic dysfunction and improve learning and memory in an APP/PS1 double transgenic mouse model of AD (Wang, Fang et al.). Morroni et al. reported the neuroprotective effects of a novel feruloyl-donepezil hybrid compound able to reduce neural damage and improve spatial cognition in mice. This approach is quite interesting, because these “chimeric” drugs take advantage of the pharmacological activities of each compound providing an efficient synergism in terms of neuroprotection.

## Author Contribution

All authors listed have made a substantial, direct and intellectual contribution to the work, and approved it for publication.

## Conflict of Interest

The authors declare that the research was conducted in the absence of any commercial or financial relationships that could be construed as a potential conflict of interest. 
